# Water-soluble benzylidene cyclopentanone based photosensitizers for *in vitro* and *in vivo* antimicrobial photodynamic therapy

**DOI:** 10.1038/srep28357

**Published:** 2016-06-21

**Authors:** Yanyan Fang, Tianlong Liu, Qianli Zou, Yuxia Zhao, Feipeng Wu

**Affiliations:** 1 Technical Institute of Physics and Chemistry, Chinese Academy of Sciences, Beijing 100190, P. R. China; 2 University of Chinese Academy of Sciences, Beijing 100049, P. R. China

## Abstract

Antimicrobial photodynamic therapy (aPDT) has been proposed to cope with the increasing antibiotic resistance among pathogens. As versatile pharmacophores, benzylidene cyclopentanone based photosensitizers (PSs) have been used in various bioactive materials. However, their reports as aPDT agents are very limited, and relationships between their chemical structures and antibacterial abilities have not been systematically discussed. Here, nine water-soluble benzylidene cyclopentanone PSs modified by polyethylene glycol (PEG), carboxylate anionic or pyridyl cationic agents are studied for aPDT. It is found that the binding/uptake abilities and aPDT effects of these PSs toward bacterial cells vary significantly when adjusting the number and position of their terminal charged groups. Though the comparable (also best) binding/uptake amounts are achieved by both cationic PS P3 and anionic PS Y1, only Y1 exhibits much more excellent aPDT activities than other PSs. Antibacterial mechanisms reveal that, relative to the favorable cell wall-binding of cationic PS P3, the anionic PS Y1 can accumulate more in the spheroplast/protoplast of methicillin-resistant Staphylococcus aureus (MRSA), which ensures its high efficient aPDT abilities both *in vitro* and *in vivo*. This study suggests the great clinical application potential of Y1 in inactivation of MRSA.

One of the most important clinical challenges of the 21^st^ century is the increasing resistance of bacteria against antibiotics^1^,^2^. For instance, the increasing methicillin-resistant *Staphylococcus aureus* (MRSA) resistance toward penicillin, methicillin and many β-lactam antibiotics has become a global crisis. At present, more than 25% of the isolated *Staphylococcus aureus* (*S. aureus*) are characterized as MRSA in nearly one-third of European countries^3^, with an even higher proportion in the United States (64.4%)^1^. What’s worse, it is reported that, there are about 700,000 lives being claimed as antibiotic-resistant infections every year at present, and by 2050, 10 million people would be die from resistant infections per year^4^,^5^. Great efforts have been made to explore novel effective antibiotics, however, it is still not enough due to the time- and money-consuming process of synthesis as well as screening of antibiotics^6^,^7^, and the horrible increasing speed of bacterial resistance.

Antimicrobial photodynamic therapy (aPDT) is proposed as an alternative methods to photo-inactivate pathogens^8^,^9^. During aPDT, the excited photosensitizer (PS) is able to react with oxygen via electron transfer (type-I) or energy transfer (type-II) to generate reactive oxygen species (ROS)^10^,^11^,^12^. Because of the rapid and effective actions of ROS as well as its multi-target nature, aPDT is less likely inducing bacterial resistance^13^,^14^. In recent years, a variety of PSs, such as porphyrin derivatives^15^, transition metal complexes^16^, conjugated polymers^17^, nanoparticles^18^,^19^,^20^,^21^ as well as novel organic chromophores^22^, have been synthesized and studied for aPDT. Many of them have proved excellent aPDT effects through *in vitro* experiments. Nevertheless, *in vivo* aPDT studies are mainly focused on a few photo-reactive substance classes, such as tetrapyrrole^9^,^23^-, fullerene^24^,^25^,^26^,^27^- and phenothiazine-based molecules^28^,^29^. For tetrapyrrole-based PSs, such as porphyrin-, phthalocyanine- and chlorine-derived molecules, they are macrocyclic organic molecules and generally share drawbacks of poor water solubility, rapid photobleaching, slow clearance, and prolonged photosensitivity in patients^30^. Although fullerenes show a long lifetime of triplet excited state to produce ROS efficiently^31^ and can exhibit little photobleaching compared to traditional tetrapyrrole-based PSs, the water solubility is the main limitation of them^32^. Actually, in both *in vitro* and *in vivo* fullerene based studies, a small amount of co-solvent, such as dimethylacetamide (DMA)^21^,^25^, is often utilized. Phenothiazine based photosensitizers, such as methylene blue (MB) and toluidine blue (TBO), have been widely used in clinical practice at this time. However, in a high mineral content environment (e.g. dentine), the aPDT effects of such PSs would be limited due to the obviously decreased binding/uptake ability toward bacterial cells^33^,^34^. Hence, lots of studies are still needed to explore novel PSs for practical applications of aPDT.

As versatile pharmacophores, benzylidene cyclopentanone based (BCB) PSs have been used in various bioactive materials. In recent, we continued to report the excellent PDT effects of nonionic and anionic BCB PSs to tumor cells^35^,^36^, and the aPDT activities of cationic BCB PSs toward both Gram-positive (Gram-(+)) and Gram-negative (Gram-(−)) bacteria^37^. In this study, to elucidate the effects of the number and position of terminal charged groups on the aPDT properties of such PSs, nine kinds of water-soluble benzylidene cyclopentanone based PSs modified by polyethylene glycol (PEG), carboxylate anionic or pyridyl cationic agents are selected to carry out a comparative research (Fig. 1a). *In vitro* experiments are based on three kinds of strains, *S. aureus*, MRSA, and *Escherichia coli* (*E. coli*). A refreshing result shows that, although the comparable (also best) binding/uptake amounts are achieved by both cationic PS **P3** and anionic PS **Y1**, only **Y1** indicates much more excellent aPDT activities than other PSs. The antibacterial mechanism is analyzed and discussed systematically. Furthermore, *in vivo* study of **Y1** against MRSA is carried out on a mouse skin infection model and an obvious curative effect is proved.

## Results and Discussion

### Solubility, lipid-water partition coefficient, UV-Vis absorption, and singlet oxygen generation

These PSs were synthesized and purified according to our previous reports^35^,^36^,^37^. The solubility in PBS, lipid-water partition coefficients (Log P) as well as UV-Vis absorption data are listed in Supplementary Table S1. It is shown that, with the exception of PS **B1**, the water solubility of these PSs is obviously increased, suggesting that this BCB scaffold is easy to be modified by various water-soluble groups with different charge properties, which not only meets the various requirements of drug administration, such as topical or intravenous injection, but also satisfies drugs with special needed charge properties. By analyzing the UV-V is absorption data, it is found that the blue shifts of symmetrical-modification structures are larger than those of asymmetrical ones. It should be due to the decreased electron donating capability of the terminal amino groups with modification. Generally, singlet oxygen (^1^O_2_) generation capability is an important index for evaluating the aPDT potential of a PS. In this work, the singlet oxygen quantum yield (Φ_Δ_) of these PSs in PBS was measured using 9,10-anthracenediyl-bis(methylene) dimalonic acid (ABDA) and Rose Bengal (RB) as ^1^O_2_ scavenger and reference, respectively^38^. The Φ_Δ_ values of **B2**, **B3**, **P1**-**P3**, and **Y1-Y3** are 0.028, 0.030, 0.036, 0.007, 0.027, 0.029, 0.028, and 0.029, respectively (Supplementary Fig. S1 and Table S1). Though these data seem low, they are relatively higher than those of porphycene-based photosensitizers (Φ_Δ_ ~ 0.004^9^), which have been demonstrated their high efficiency to photo-inactivate *Candida* both *in vitro* and *in vivo*. It suggests that such low Φ_Δ_ values in PBS are enough for aPDT.

### Binding/uptake of PSs by bacterial cells

**Figure 1:**
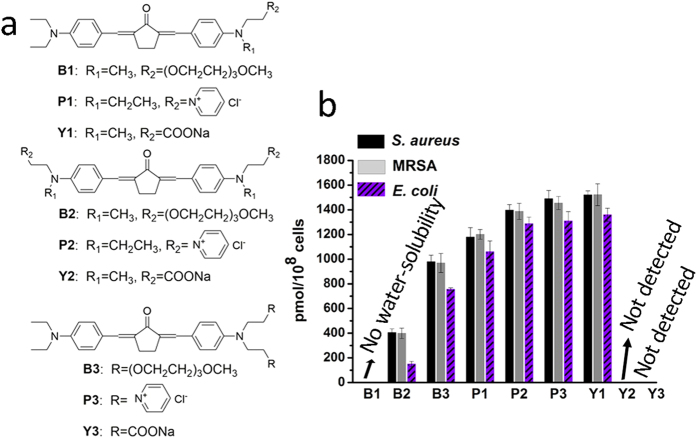
(**a**) Chemical structures of water-soluble benzylidene cyclopentanone based photosensitizers. (**b**) The binding/uptake amounts of PSs by *S. aureus*, MRSA, and *E.coli*. The error bars denote standard deviation of three replicates.

The binding/uptake amounts of PSs by bacterial cells were conducted with 10 μM of PSs to not only ensure adequate fluorescence signals to be detected but also keep the activity of bacterial cells. After incubating bacteria with PSs for 1 hour in dark, the bacterial cells were centrifuged, washed, and then lysed. Since PS **B1** has not enough water solubility, it is excluded in this test. The results are shown in Fig. 1b. The binding/uptake amounts of PSs **Y2** and **Y3** by all strains are too low to be detected, while the data of other PSs exhibit the same order in different strains as **Y1 **> **P3 **> **P2 **> **P1 **> **B3 > B2**. For example, the uptake amounts of **B2**, **B3**, **P1**, **P2**, **P3**, and **Y1** by MRSA are 399, 969, 1201, 1387, 1455, and 1522 pmol/10^8^ cells, respectively, and by *E. coli*, the corresponding data are 150, 756, 1060, 1288, 1310, and 1360 pmol/10^8^ cells, respectively. In general, the membrane surfaces of microbial pathogens, including bacteria and fungi, are negatively charged under physiological conditions^39^, so that positively charged PSs can bind to their surfaces via electrostatic interaction, which not only ensures the high binding/uptake amounts of cationic PSs but also provides them with inactivation abilities induced by the destabilization and interruption effects of the bound cations toward negative cell walls^40^. For neutrally and negatively charged PSs, without the merit of electrostatic interaction, their binding/uptake efficiency by bacteria are commonly reported to be inferior to cationic ones, especially by the Gram-(−) strains, whose cell wall has an extra densely organized outer layer^41^. Here, cationic PSs **P1**, **P2**, and **P3** achieve higher binding/uptake amounts than those of **B2**, **B3**, **Y2**, and **Y3**, indicating the obvious advantage of their positive charges. But, interestingly, the anionic PS **Y1** possesses the highest binding/uptake amounts among all PSs. In fact, a similar result had been reported by Demidova *et al*. that the two negatively charged RB exhibited a higher binding/uptake amounts than that of one positively charged Toluidine blue-O (TBO) in *E. coli*^42^. However, due to the quite different molecular structures of RB and TBO, no in-depth discussion was conducted. In this study, based on a same molecular scaffold for these BCB PSs, it supplies a chance to explore the deep reason. Based on Lipinski’s “rule of five” for desirable drugs^43^, we speculate that the multiple advantages of concise chemical structure, low molecular weight (Mw, <500 Dalton) as well as high Log P within limitation (<5) endow **Y1** the excellent binding/uptake abilities, and more detailed discussions will be carried out associated with the following experimental results . In addition, the binding/uptake abilities of PSs by Gram-(−) *E. coli* are all relatively inferior to those of Gram-(+) strains. It is reasonable due to the barrier effect of the extra outer layer in *E. coli*.

### *In vitro* antimicrobial photodynamic therapy

**Figure 2:**
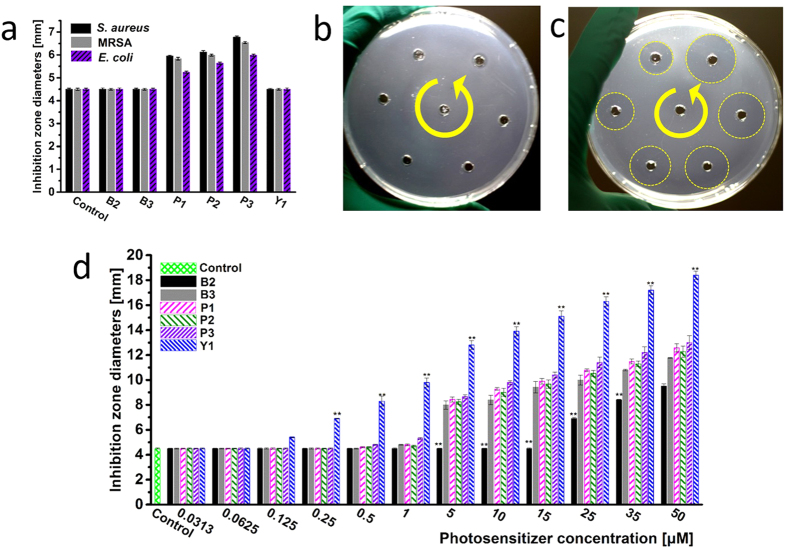
The data of inhibition zone diameters with different PSs at different concentrations. (**a**) PSs against all strains in dark with a concentration of 10 μM. Image of **Y1** against MRSA at different concentrations of 5, 10, 15, 25, 35, and 50 μM, respectively, (**b**) In dark, (**c**) With a 532 nm laser (50 mW cm^−2^, 10 min, 30 J cm^−2^). (**d**) PSs against MRSA with a 532 nm laser (50 mW cm^−2^, 10 min, 30 J cm^−2^) at different concentrations. (**P < 0.05 compared with the corresponding data of other PSs).

To comparing the antibacterial abilities of these PSs, the inhibition zone test was conducted firstly. Because the binding/uptake amounts of PSs **Y2** and **Y3** by bacterial cells are not detectable, combing with their undesirable results in preliminary inhibition zone test, PSs **Y2** and **Y3** are excluded in further studies. The results of inhibition zone tests are shown in Fig. 2. In dark, neutral PSs **B2** and **B3**, and anionic **Y1** have no obvious antibacterial activities at their concentrations of 10 μM, while the inactivation ability of cationic PSs **P1**–**P3** is obvious (Fig. 2a), confirming that cationic PSs have electrostatic interaction-based dark toxicity. When irradiating bacterial plates with a 532 nm laser (50 mW cm^−2^, 10 min, 30 J cm^−2^), all PSs exhibits varying degrees of aPDT effects. The order of the inhibition zone diameters of these PSs against three strains is consistent, being **Y1 ≫** **P3 **> **P1 **> **P2 **> **B3 ≫** **B2**. It is entirely unexpected that **Y1** indicates the most excellent aPDT activity (Fig. 2c,d, Supplementary Figs S2 and S3), which is even much better than that of cationic PS **P3**. The results are further confirmed by the quantitative evaluation of the minimum inhibitory concentration (MIC) tests. As shown in Table 1, the MIC values of PS **Y1** against *S. aureus*, MRSA, and *E. coli* are 0.0625, 0.0625 and 0.125 μM, respectively, much lower than those of other PSs (Supplementary Fig. S4). Considering the comparable Φ_Δ_ values and very similar binding/uptake amounts of **Y1** and **P3**, it seems that factors other than ^1^O_2_ generation play role in their significant discrepancy of aPDT effects.

### Zeta potential tests and intracellular distribution

**Figure 3:**
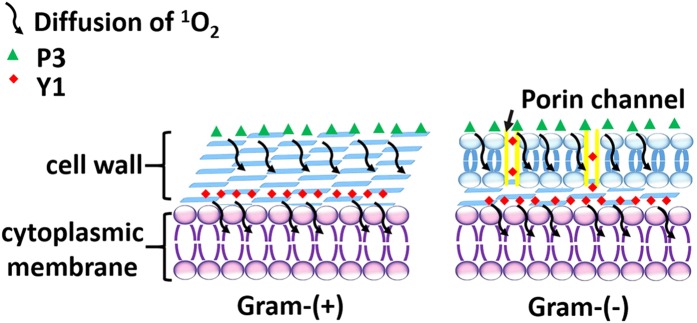
The schematic diagram to illustrate the photodynamic inactivation mechanisms of PSs against Gram-(+) and Gram-(−) bacteria.

Zeta potentials of *S. aureus*, MRSA and *E. coli* with or without PSs were tested after washing bacteria for three times. Three PSs, **B3**, **P3**, and **Y1**, which show the best aPDT effects among their analogs are chosen as models with different charge properties. As shown in Table 2, **B3**, and **Y1** exhibit negligible effects on zeta potentials of bacteria cell walls, while the negative charge densities of all strains obviously decreased in the presence of **P3**, hinting the surface binding of **P3** with bacterial cell walls as well as the deeper diffusion of **Y1**. Considering the short lifetime and the action radius of ^1^O_2_ in biologic systems^44^,^45^ (<0.04 ms and <0.02 μm, respectively^46^) as well as the cell walls’ thickness (about 15 ~ 80 nm and 10 ~ 15 nm for Gram-(+) and Gram-(−) bacteria^13^, respectively), the ^1^O_2_ produced by surface bound **P3** have to travel a longer distance to touch the cell membrane (the main target of aPDT^47^) while most ^1^O_2_ produced by **Y1** can play their antibacterial roles in the inner of bacteria cell walls locally (Fig. 3). As a result, the aPDT effect of **P3** would be significantly discounted relative to that of **Y1**. This point can be further supported by the intracellular distribution^48^,^49^ of PSs in MRSA as shown in Table 3. Relative to ~50% of **B3** and **P3** located in cell walls, more than 70% of **Y1** were found in spheroplast/protoplast.

Considering all results above, here, it is supposed that, the synergy effects of concise chemical structure, suitable Log P value as well as negative charge endow PS **Y1** the best aPDT effects. It’s well known that lipophilic character of a PS can increase its affinity toward bacteria cells. However, though PSs **B2**, **B3**, and **Y1** have similar Log P (~3.0), their binding/uptake amounts are quite different which indicates that lipophilic character is just one factor affecting binding/uptake abilities and further aPDT effects of PSs, but not the dominate one. As H. Nikaido reported, the Mw had great effect on the uptake efficiency of solutes by bacteria^50^. The Mw of **Y1** is the lowest one among all PSs in this study. Obviously, the Mw should be also one influencing factor. However, it is worth to be noted that, although the Log P and Mw of **B2** are comparable with those of **B3**, their binding/uptake amounts are still quite different. It seems their different molecular configurations play a key role. Finally, by analyzing the similar binding/uptake amounts of PS **P3** and **Y1** as well as their different intracellular distribution percentage in cell wall and spheroplast/protoplast, it is considered that, though positive charge induced electrostatic interaction is benefit to the binding of a cationic PS onto the negatively charged bacterial cell walls, it may also restrain the further entry of the PS, while a negative charged PS can escape this restriction to readily diffuse into the cell.

As shown in Supplementary Fig. S5, the IC_50(light)_ of **Y1** (the concentration of **Y1** to achieve 50% growth inhibition of cells under 532 nm irradiation with the same dosage as determined MIC values in this study) toward mammalian cell L929 is 4.1 μM. It is ≥32 times of the MIC of **Y1** to the three strains, indicating **Y1** has high selective photodynamic inactivation to bacteria over mammalian cells. What’s more, as shown in Supplementary Fig. S6, PS **Y1** is less hemolytic active toward human red blood cells (hRBC) when compared with those of PSs **B3** and **P3**. The hemolysis percentage of **Y1** is less than 5% with a concentration up to 40 μM, indicating its good biological safety. In addition, compared with PS **P3**, the uptake of **Y1** by mammalian cells is more efficient (Supplementary Figs S7 and S8). As some *S. aureus* are supposed to survive the standard antibiotic treatment by “hiding” in mammalian cells^51^, it is urgent to explore novel antibacterial agents, especially for potential ones that having selective inactivation to intracellular pathogens. In this study, the above features fit **Y1** with a great potential to inactive pathogens “hiding” in host cells.

### *In vivo* antimicrobial photodynamic therapy against MRSA

**Figure 4:**
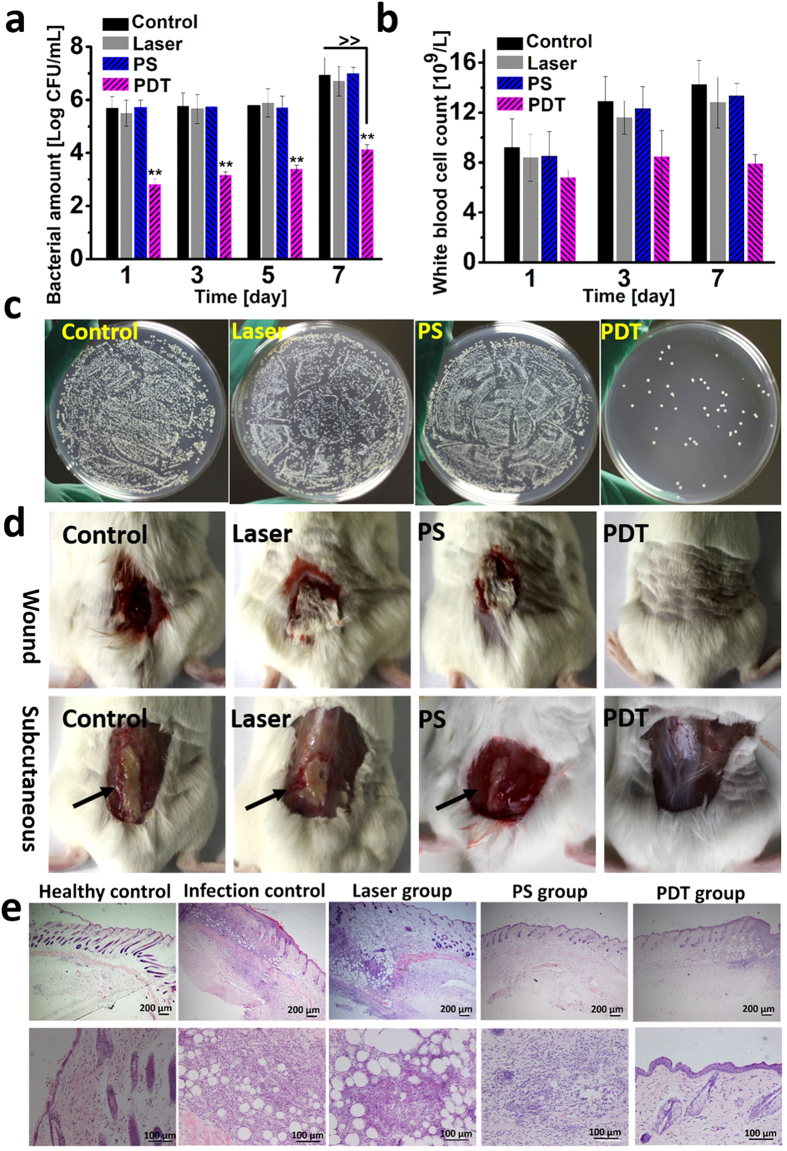
(**a**) Bacterial amount of the control, laser, PS, and PDT group at different days after treatment, data of days 1, 3, and 5 were obtained by isolating MRSA from the mice skin. ≫means the data were acquired from tissue serous (0.01 g/mL) on the 7^th^ days, “**” indicates statistical significance relative to the control group, p < 0.05. (**b**) The white blood cell count (WBC) of different groups at different days after PDT treatment. (**c**) Plate photographs of bacterial amount of tissue serous (0.01 g/mL), 100-fold dilution respectively. (**d**) The wound healing results and the subcutaneous tissue infection results of the control, laser, PS, and PDT groups after 7 days of treatments. (**e**) Histological analysis of tissues in healthy control, infection control, laser, PS, and PDT groups.

Based on the *in vitro* studies above, the potential of PS **Y1** to fight against MRSA *in vivo* was carried out. In this study, a mouse skin wound infection model was designed. Adult male ICR mice, 6–8 week old, were wounded and infected with 50 μL 10^8^ CFU/mL suspension of MRSA. Cytotoxicity results showed that, at the concentration of 2.5 μM, **Y1** had negligible dark- and photo-cytotoxicity toward L929 cells (cell viability >90%) (Supplementary Fig. S5). Therefore, 50 μL of photosensitizer solution (2.5 μM) or PBS were applied to the wound after 12 hours of infection, followed by irradiation with a 532 nm laser (50 mW cm^−2^, 10 min, 30 J cm^−2^). As data shown in Fig. 4a, compared with other groups, more than 99.5% bacterial cells are inactivated in PDT group and no obvious bacteria recrudescent proliferation is found in the following days, suggesting the viability of the aPDT treatment. Meanwhile, the counts of the white blood cells (WBC) at different days are also detected to observe the inflammation in mice (Fig. 4b). It shows that, though the WBC has a slight rise on the 3^rd^ day in PDT group, the index basically stabilizes in normal conditions (5.1 ~ 11.6 × 10^9^/L) over time. Nevertheless, inflammation in other groups became more and more serious. On the 7^th^ day after treatment, the number of MRSA in the infected tissues was counted. Tissue serous (0.01 g/mL) with different dilution multiples was utilized to spread plate, and the colony images in different groups with 100-fold dilution were shown in Fig. 4c. Results show that about 99.8% of MRSA are inactivated in PDT group, while no significant MRSA death is detected in other groups. Additionally, as shown in Fig. 4d, the PDT-treated mice have significant advantage in wound healing and negligible infection in subcutaneous tissue over other groups. The results of histological analysis of skin tissues reveal that, comparing with the healthy tissues, the skin structure in infection control group is loose with edema, blood vessels in the dermis are congested, and have lots of lymphocytes infiltration and obvious inflammatory reaction (Fig. 4e). In the laser and PS group, lymphocytes infiltration and congestion are found in the dermis, suggesting the inflammation. As for the PDT group, inflammatory reaction seems negligible that indicating the curative effect of aPDT.

## Conclusion

In conclusion, the aPDT properties of nine kinds of water-soluble benzylidene cyclopentanone based PSs modified by PEG, carboxylate anionic or pyridyl cationic agents were comparatively studied. The results show that the number and position of the terminal charged groups have a great influence on the binding/uptake abilities as well as aPDT effects of these PSs. In addition, it seems that the distribution of a PS within bacterial cells also has significant effect on its aPDT activity. Though the electrostatic interaction is beneficial to cationic PSs to bind easily to the surface of bacterial cell walls, it may also restrict their further entry. On the contrary, when anionic PSs are endowed equivalent binding/uptake capability, they can diffuse deeper into bacterial cells to play antibacterial roles more efficiently. Here, **Y1** is a very good example. Moreover, PS **Y1** also provides the possibility of selectively inactivating pathogens “hiding” in mammalian cells without damage to host tissues. After carrying out *in vivo* experiments against MRSA, the great aPDT potential of **Y1** in clinical usage is also demonstrated. This work provides a new understanding of anionic PSs and may inspires the corresponding aPDT studies.

## Methods

### Binding/uptake amounts of PSs

For the binding/uptake test, 10 μM of PSs were co-incubated with bacterial suspensions (bacteria density ~10^8^ CFU/mL) at 37 °C for 1 hour in dark, and the bacteria pellets were obtained by centrifugation at 6000 rpm for 10 min. After washing the achieved pellets with PBS for three times, the bacteria were lysed with lysozyme (100 μg/mL, 1 hour) and sonication (at 37 °C, 2 hours), and the fluorescence signals of the supernatant of the lysed bacterial solution were utilized to calculate the binding/uptake amounts of PSs.

### Agarose diffusion assay

Agarose diffusion assay was carried out in accordance to Lehrer *et al.*^52^. 100 mL of agarose at the temperature of 50 °C was mixed with 3 mL of bacterial cell suspensions (~10^5^ CFU/mL) and then poured immediately into a sterile Petri dish. After cooling the dish at room temperature for 30 min, the sterile puncher was utilized to punch wells in which 20 μL of PBS solutions with different concentrations of PSs were added. All the plates were incubated upright at 37 °C for 1 hour. After that, the control group was incubated in the incubator continuously, and the experimental group was irradiated by a 532 nm laser (50 mW cm^−2^, 30 J cm^−2^) for 10 min. After another 24 hour of incubation at 37 °C, the antimicrobial activity was quantitated by measuring the diameter of inhibition zones on the opaque background of bacterial growth.

### The minimum inhibitory concentration (MIC) test

The MIC test was conducted using the modified resazurin method^53^. Detailed experimental operation was in accordance to our previous report^37^. The final concentration of bacterial cell suspensions was 5 × 10^5^ CFU/mL, and the PS concentrations ranged from 0.0313 to 32 μM. A 532 nm laser (50 mW cm^−2^, 30 J cm^−2^, 10 min) was used to irradiate 96-well plates. MIC values were recorded and calculated after 18 hours of incubation at 37 °C.

### Zeta potentials test

5 μM of PSs were cultured with bacterial suspensions of ~ 10^8^ CFU/mL for 1 hour in dark at 37 °C. Then, the suspensions were centrifuged and washed for three times. Malvern Zetasizer 3000HS (Malvern Instruments Ltd.) was used to measure the zeta potentials.

### Mouse model of skin wound infected with MRSA

In the experiment, adult male ICR mice (obtained from Vital River Laboratory Animal Technology Co. Ltd., Beijing), aged 6–8 weeks, were utilized. The protocols used in animal experiments were approved by the Laboratory Animal Centre of Peking University, China. The methods were carried out in accordance with the relevant guidelines, including any relevant details. 3% pentobarbital sodium was injected into intraperitoneal to anesthetize mice before the dorsal surfaces being shaved. A skin site with a 1 cm × 1cm wound was made by sterile syringe needle with the diameter of 0.4 mm. The depth of puncture is about 1 mm marked on the needle by marking pen. After wounded, 50 μL of MRSA suspensions (~10^8^ CFU/mL) was inoculated over the wound site. To verify mice skin infection model, bacterial culture and biochemical identification were performed. Mice were randomly divided into four groups, each group had six mice.

### *In vivo* photodynamic treatment and infection detection

After infection, these four groups of mice were treated, respectively. 2.5 μM of PS **Y1** was applied to the wound because the dark- and light-cytotoxicity at this concentration were both negligible. 50 μL solution of PBS for control group, 50 μL solution of PBS followed by illumination with a 532 nm laser (30 J cm^−2^, 10 min) for laser group, 50 μL solution of **Y1** (2.5 μM) for PS group, and 50 μL solution of **Y1** (2.5 μM) followed by illumination with a 532 nm laser (50 mW cm^−2^, 30 J cm^−2^, 10 min) for PDT group. All animals were treated single time. To ensure no adverse reactions, mice were checked twice daily during infection and treatment. After PDT treatment, on the 24 hours, 3 days, 5days and 7days, clinical examination, bacterial culture and biochemical identification were performed to verify mice skin infection model. For bacterial culture, a sterile cotton rod swapped on the wound and steeped in 2 mL sterile PBS for 5 min, then 200 μL PBS solution with different dilution multiples were inoculated using plate smearing method. Colonies were enumerated and reported by the method of agar plate counting. On the 7^th^ day, wound tissues were taken sterilely and their corresponding tissue serous were obtained using the tissue pulp apparatus (0.01 g/mL). Bacteria counts of the tissue serous were conducted using the same method above. The white blood cell count (WBC) was also detected using MEK722 to observe the inflammation in mice.

### Statistical analysis

One-way analysis of variance (ANOVA) test assisting with SPSS 16.0 (SPSS Inc., Chicago, IL) was utilized to evaluate the multi-groups comparisons of the means. *P* < 0.05 represented the statistical significance for all tests.

## Additional Information

**How to cite this article**: Fang, Y. *et al.* Water-soluble benzylidene cyclopentanone based photosensitizers for *in vitro* and *in vivo* antimicrobial photodynamic therapy. *Sci. Rep.* **6**, 28357; doi: 10.1038/srep28357 (2016).

## Supplementary Material

Supplementary Information

## Figures and Tables

**Table 1:** The results of minimum inhibitory concentration (MIC) tests, unit: μM.

Strains	B2	B3	P1	P2	P3	Y1
*S. aureus*	>32	2.0	2.0	2.0	1.0	0.0625
MRSA	>32	2.0	2.0	2.0	1.0	0.0625
*E. coli*	>32	4.0	4.0	4.0	2.0	0.125

**Table 2:** The results of zeta potentials, unit: mV.

Strains	*S. aureus*	MRSA	*E. coli*
Blank	−30.5 ± 4.3	−29.7 ± 4.1	−31.3 ± 3.7
B3	−31.1 ± 2.9	−30.9 ± 3.3	−30.9 ± 3.1
P3	−21.2 ± 3.7	−19.8 ± 2.1	−20.3 ± 2.6
Y1	−31.3 ± 4.4	−30.6 ± 3.6	−31.5 ± 2.4

The bacterial cells were incubated with or without 5 μM of PSs, data were expressed as means ± standard deviation of three independent experiments.

**Table 3:** Intracellular distribution of PSs.

Compounds	S/P (%)	W (%)
B3	46.4 ± 7.3	53.6 ± 7.3
P3	50.6 ± 4.8	49.4 ± 4.8
Y1	73.6 ± 2.4	26.4 ± 2.4

PSs in the cell wall (W) and the spheroplast/protoplast (S/P) after 1 hour incubation with MRSA, data were expressed as means ± standard deviation of three independent experiments.
