# Supplementary material for: Water-soluble benzylidene cyclopentanone based photosensitizers for in vitro and in vivo antimicrobial photodynamic therapy
Source: Sci Rep. 2016 Jun 21;6:28357. doi: 10.1038/srep28357 (PMC4914934; doi:10.1038/srep28357)
Supplement: Supplementary Information [file srep28357-s1.pdf]

Supplementary Information
for

Water-soluble benzylidene cyclopentanone based photosensitizers for *in vitro* and *in vivo* antimicrobial photodynamic therapy

Yanyan Fang[a, b], Tianlong Liu[a], Qianli Zou[a], Yuxia Zhao[a,*] & Feipeng Wu[a,*]

[a] Technical Institute of Physics and Chemistry, Chinese Academy of Sciences, Beijing 100190, P. R. China; [b] University of Chinese Academy of Sciences, Beijing 100049, P. R. China.

[*]Corresponding author: For Yuxia Zhao: Tel, +86 10 82543569; Fax, +86 10 82543491; E-mail, yuxia.zhao@mail.ipc.ac.cn. For Feipeng Wu: Tel, +86 10 82543569; Fax, +86 10 82543491; E-mail, fpwu@mail.ipc.ac.cn.

Materials

Staphylococcus aureus (*S. aureus*) and *Escherichia coli* (*E. coli*) were obtained from Antimicrobial Test Center, Technical Institute of Physics and Chemistry, Chinese Academy of Sciences. Methicillin-resistant *Staphylococcus aureus* (MRSA) was a gift from Department of Laser Medicine, Chinese PLA General Hospital. Human red blood cells (hRBC) were donated by volunteers. 9,10-anthracene-diyl-bis (methylene)dimalonic acid (ABDA) were purchased from Sigma Aldrich

Chemical Company. Phosphate buffered saline (PBS) solution (pH=7.4) and Dubach's modified Eagle's medium (DMEM containing 4.5 g/L glucose, 100 unit/mL penicillin, 100 μg/mL streptomycin) were from Beijing Solarbio Science & Technology Co. Ltd. Cell Counting Kit-8 (CCK-8) were from Beyotime Institute of Biotechnology. Fetal bovine serum (FBS) was from Hangzhou Sijiqing Co. Ltd. Luria Bertani (LB) broth, Mueller-Hinton (MH) broth and nutrient agar were from Qingdao Hope Biol-Technology Co. Ltd.

Instruments

UV-Vis spectra were measured by a Hitachi U-3900 spectrophotometer. Steady-state fluorescence was carried out on a Hitachi F-4500 spectrometer. Zeta potentials were measured in PBS suspensions on a Malvern Zetasizer 3000HS (Malvern Instruments Ltd.). The white blood cell count was obtained by blood analyzer MEK722. Light source for aPDT was a 532 nm diode pumped solid-state laser (Millennia, Spectra-Physics, continuous wave). Thermo MK3 plate reader was used to measure the absorbance of the solution in 96-well plates. The confocal laser scanning microscope (CLSM) images were detected on Olympus FV1000.

Singlet oxygen quantum yield

The singlet oxygen quantum yield (Φ_Δ) was determined using Rose Bengal (**RB**) as the reference with a yield of 0.75 in PBS. A mixed solution of a photosensitizer and **ABDA** was prepared. In the solution, the concentration of the photosensitizer was adjusted to possess the same absorbance (1.0) at 532 nm and the absorbance of **ABDA** at 402 nm was also adjusted to 1.0. In the experiment, the solution was stirred vigorously to ensure the saturation of air. When the solution was irradiated by a 532 nm diode laser, the bleaching of the absorption band of **ABDA** at 402 nm was monitored. The solution of **ABDA** alone was also irradiated to diminish the errors origin from the photo-activation. The Φ_Δ of each compound was calculated by the following equation:

$$\Phi_{\Delta}^{S} = \frac{k_S}{k_R} \times \Phi_{\Delta}^{R}$$

Where K is the slope of the photodegradation rate of **ABDA**, S means the sample, R means the reference, and Φ_{Δ}^{R} is the singlet oxygen quantum yield of the reference.

Bacterial growth

All apparatus and materials were autoclaved, and all operations were handled under a sterile condition. Three strains were revived with Luria Bertani (LB) broth and nutrient agar at 37 °C for 24 hours. The optical density at 600 nm (OD_{600}) was monitored to determine the density of bacterial cells.

Intracellular distribution of PSs in bacterial cells[1,2]

The subcellular distribution of photosensitizers was evaluated after fractionation of treated bacterial cells. After 1 hour incubation of 10^8 CFU mL^{-1} bacteria with photosensitizers at 37 °C with shake, the cell suspensions were centrifuged at 6000 rpm for 10 min. A pellet of bacterial cells was re-suspended in 2 mL Tris-HCl (0.05 M, pH 7.2) solution containing, 0.5 M sucrose, 0.01 M magnesium acetate and 1 mg lysozyme, and lysed in dark for another 1 hour. Then the spheroplasts and protoplasts of bacterial cells were separated from the cell walls by a reported centrifugation based method[1,2]. After re-centrifugation, the pellets containing either spheroplasts or the protoplasts were re-suspended in 4 mL SDS aqueous solution (2%), while the supernatant containing the cell wall were diluted by 2 mL SDS aqueous solution (4%). The UV-visible absorption spectra of the resulting solutions were determined to estimate the intracellular distribution of dye molecules.

Dark- and photo-cytotoxicity of Y1 toward L929 cells

For the dark cytotoxicity, L929 cells were dispersed and cultured in 96-well plates for 24 h. Then, 100 μL culture medium containing different concentrations of PS were used to replace the original culture solution. After another 24 h of incubation, the cell viability was determined by CCK-8 assay. For photo-cytotoxicity, a 532 nm laser (30 J cm^{-2}, 10min) was used to irradiate the cells after incubating them with **Y1** for 4 h. After irradiation, the cells were incubated in dark for another 24 h before testing. Cells without PSs were also treated with the same laser and were used as the 100% cell survival base line.

Hemolysis assay

The hemolysis assay was conducted according to the report of Wang *et al*[3]. Methods were carried out in accordance with the relevant guidelines, including any relevant details. Briefly, fresh human red blood cells (hRBC) with EDTA were washed with PBS for five times and collected by centrifugation at 6000 r min^{-1} for 5 min. After that, hRBC was diluted via a volume ratio $V_{(hRBC):}V_{(PBS)}$=1:9, and PS-PBS solutions were prepared with the PS concentration ranging from 0.313 to 80 μM. In a 12-well plate, 450 μL of both PS-PBS solution and hRBC dilution were added to one well to achieve the final erythrocyte percentage of 5% (v/v) and PS concentration ranging from 0.157 to 40 μM. For the positive and negative control, 450 μL of 1% Triton-X100 and PBS were used respectively to replace PS-PBS solution. After 4 hours of incubation at 37 °C with shake, the UV-Vis absorptions of samples were detected to calculate the hemolysis percentage. Each assay was replicated for three times.

$$\text{Hemolysis percentage (\%)}=\frac{\text{OD(test)-OD(negative control)}}{\text{OD(positive control)-OD(negative control)}}\times 100\%$$

Subcellular localization of PSs in HepG2 cells

To detect the subcellular localization, a confocal laser scanning microscope (Nikon AIR MP) was used. HepG2 cells were incubated in a cover glass chamber (diameter 35 mm) for 24 h to reach a density of 10^5 cells per well. Medium containing 10 μM PSs was used to replace the original culture solution. After 8h incubation, the chambers were washed with PBS three times. Then the culture medium with 100 nM Mito-tracker green was used to incubate the cells for 20 min and the chambers were washed with PBS again. A light of 488 nm was used to excite Mito-tracker green, while the PSs were excited by a 561 nm light.

Cellular uptake by HepG2 cells[4]

Cellular uptake profiles were determined by lysis method plus with fluorescence spectrometer. Briefly, the cells in 12-well plates were incubated with 10 μM photosensitizers for certain time, washed three times with PBS, added with 200 μl lysis buffer (20 mM Tris, pH 7.5, 150 mM NaCl, 1% Triton X-100), put on ice for 10 min, then added with 1 ml PBS. The fluorescence signal of the final solution was measured. Standard curves were obtained by dissolving photosensitizers in lyzed solution of blank cells. The photosensitizer concentration was converted to intracellular concentration by assuming that the volume of each HepG2 cell was 2.83×10^{-9} ml and the photosensitizer was distributed equally within the cell[5].

**PSs**	Solubility (mg mL^{-1})	Log P	λ_{max} (nm)	ε_{max} (10^4 M^{-1} cm^{-1})	Φ_Δ
**B1**	-	-	-	-	-
**B2**	2.8	3.0	508	5.0	0.028
**B3**	2.6	3.0	511	4.5	0.030
**P1**	4.3	1.8	501	4.1	0.036
**P2**	> 5	-1.0	489	5.9	0.007
**P3**	> 5	-0.1	498	4.5	0.027
**Y1**	0.8	2.9	512	3.7	0.029
**Y2**	> 5	0.3	507	6.2	0.028
**Y3**	> 5	-0.7	516	4.8	0.029
λ_{max} is the UV-Vis absorption maximum in PBS; ε_{max} is molar absorption coefficient at λ_{max}; Φ_Δ is singlet oxygen quantum yield measured by photochemical trap method in PBS. The data of solubility, Log P, λ_{max}, and ε_{max} are from our previous work in ref. 6-9.					

Supplementary Table S1. Solubility in PBS, lipid-water partition coefficient (Log P), UV-Vis absorption, and singlet oxygen generation

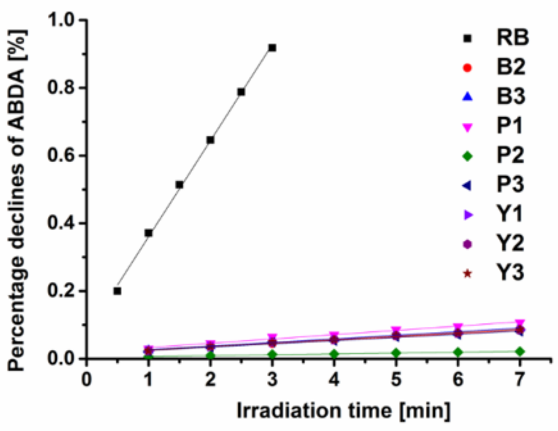

Supplementary Figure S1. The percentage declines of ABDA vs. irradiation time under a 532 nm laser.

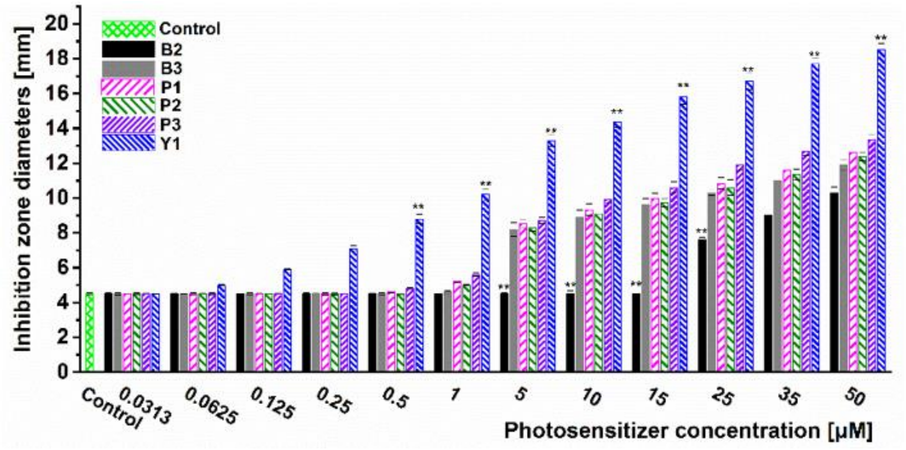

Supplementary Figure S2. The inhibition zone diameters of *S. aureus* with different PSs at different concentrations with a 532nm laser (50 mW cm^{-2}, 10 min, 30 J cm^{-2}). (** $P < 0.05$ compared with the corresponding data of other PSs).

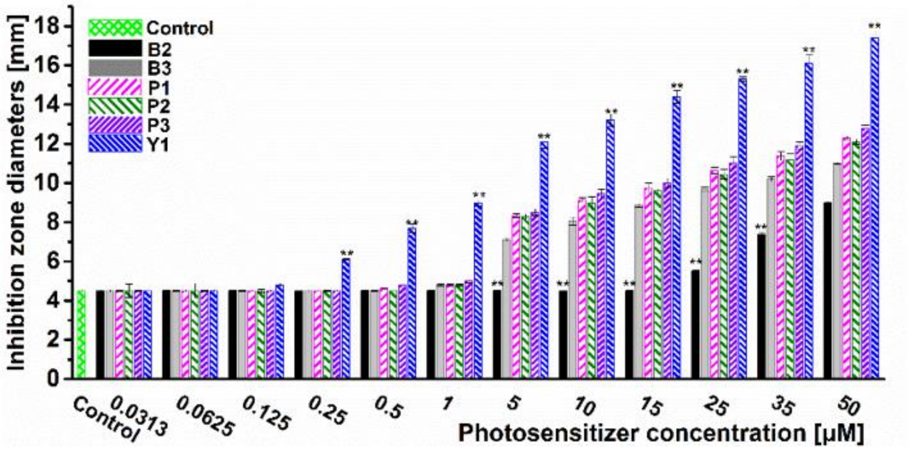

Supplementary Figure S3. The inhibition zone diameters of *E. coli* with different PSs at different concentrations with a 532nm laser (50 mW cm^{-2}, 10 min, 30 J cm^{-2}). (** $P < 0.05$ compared with the corresponding data of other PSs).

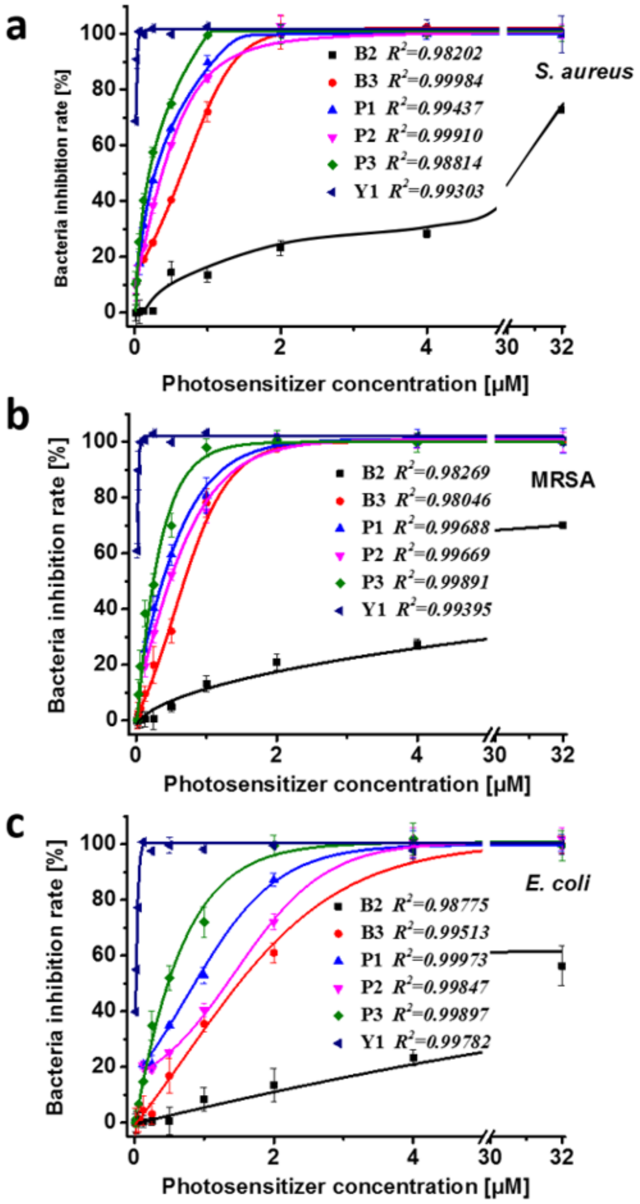

Supplementary Figure S4. Relative inhibition rate of *S. aureus*, MRSA, and *E. coli*, respectively.

The error bars denote standard deviation of three replicates.

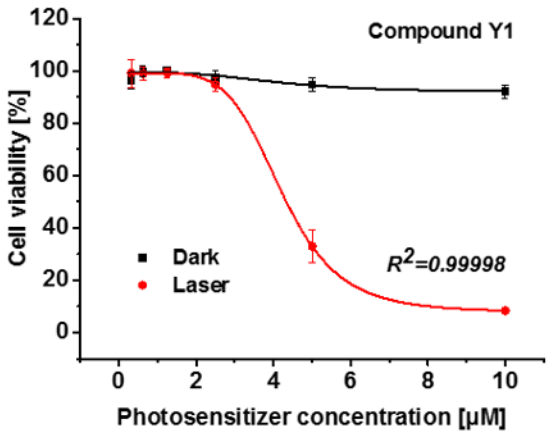

Supplementary Figure S5. Cytotoxicity of Photosensitizer **Y1** toward L929 cells in dark or with a 532 nm laser (50 mW cm^{-2}, 10 min, 30 J cm^{-2}) after 4 hours of uptake. The error bars denote standard deviation of three replicates.

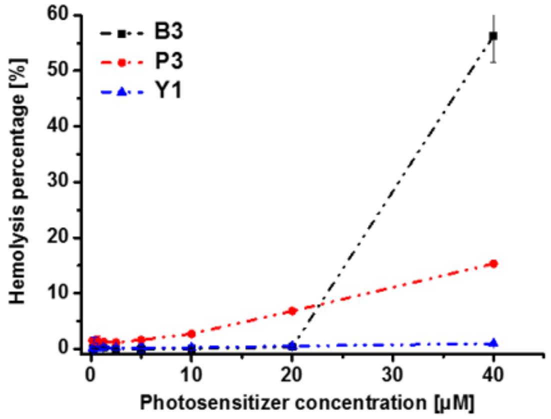

Supplementary Figure S6. Hemolytic activities of the examined photosensitizers at varied concentrations. The error bars denote standard deviation of three replicates.

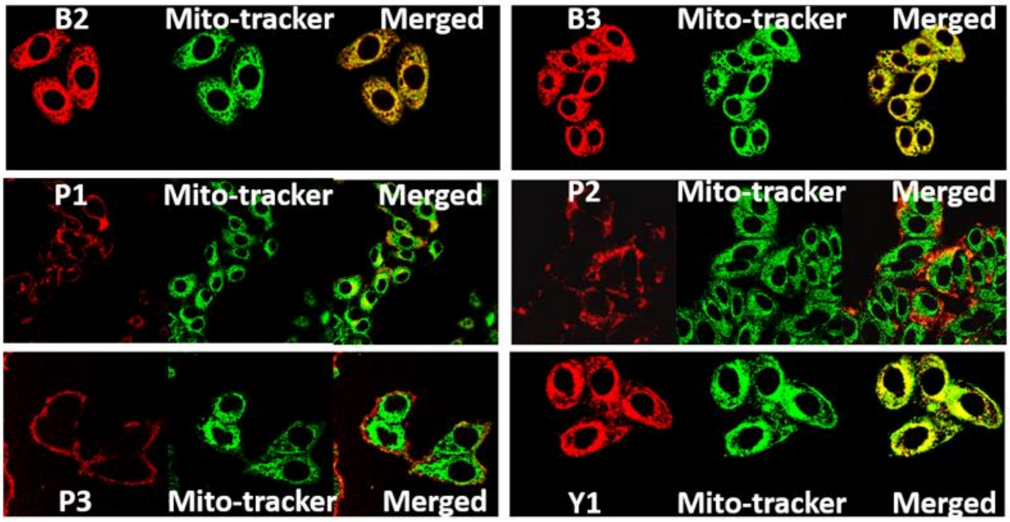

Supplementary Figure S7. Confocal fluorescence images obtained by incubating HepG2 cells with 10 μM PSs for 8 h and 100 nM Mito-tracker green for 20 min.

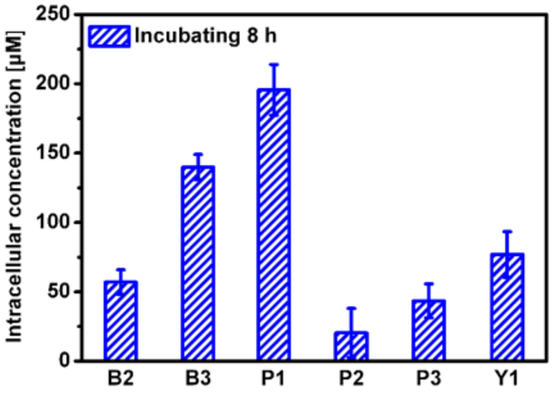

Supplementary Figure S8. Intracellular concentrations of PSs after incubating HepG2 cells for 8 h with 10 μM photosensitizers. The error bars denote standard deviation of three replicates.

References

1 Li, K. *et al.* A bivalent cationic dye enabling selective photo-inactivation against gram-negative bacteria. *Chem. Commun.* **51**, 7923-7926 (2015).

2 Merchat, M., Spikes, J. D., Bertoloni, G. & Jori, G. Studies on the mechanism of bacteria photosensitization by meso-substituted cationic porphyrins. *J. Photochem. Photobiol.*, B **35**, 149-157 (1996).

3 Wang, H. *et al.* Antibacterial activity of geminized amphiphilic cationic homopolymers. *Langmuir* **31**, 13469-13477 (2015).

4 Zou, Q. *et al.* Effective two-photon excited photodynamic therapy of xenograft tumors sensitized by water-soluble bis(arylidene)cycloalkanone photosensitizers. *J. Med. Chem.* **58**, 7949-7958 (2015).

5 Bondarava, M. Hypertonicity-induced cation channels in hepg2 cells: Role in proliferation and putative molecular correlates. *Max Planck Institute of Molecular Physiology & Behavior, Bochum* (2007).

6 Yang, W. *et al.* Carboxylate modified benzylidene cyclopentanone dyes for one- and two-photon excited photodynamic therapy. *J. Photochem. Photobiol., A* **222**, 228-235 (2011).

7 Zhao, Y. X. *et al.* Polyethylene glycol-functionalized benzylidene cyclopentanone dyes for two-photon excited photodynamic therapy. *Org. Biomol. Chem.* **9**, 4168-4175 (2011).

8 Fang, Y., Liu, T., Zou, Q., Zhao, Y. & Wu, F. Cationic benzylidene cyclopentanone photosensitizers for selective photodynamic inactivation of bacteria over mammalian cells. *RSC Adv.* **5**, 56067-56074 (2015).

9 Fang, Y. Y. *et al.* Water-soluble benzylidene cyclopentanone photosensitizers for two-photon excited photodynamic therapy. *Proc. of SPIE* **8923**, 892319-8 (2013).
